# Beneficial effects of time-restricted fasting on cardiovascular disease risk factors: a meta-analysis

**DOI:** 10.1186/s12872-024-03863-6

**Published:** 2024-04-16

**Authors:** Zhengqi Qiu, Emma Yun Zhi Huang, Yufei Li, Ying Xiao, Yancheng Fu, Jun Du, Juntao Kan

**Affiliations:** 1 Faculty of Medicine, Macau University of Science and Technology, Avenida WaiLong, Taipa, 999078 Macau China; 2 Department of Biochemistry and Molecular Biology, School of Basic Medical Sciences, Health Science Center, Shenzhen University, Shenzhen, 518060 China; 3 Nutrilite Health Institute, Shanghai, China

**Keywords:** Cardiovascular disease, Time-restricted fasting, Rhythm, Meta-analysis, Weight loss

## Abstract

**Background:**

Cardiovascular disease continues to be a leading cause of mortality worldwide, highlighting the need to explore innovative approaches to improve cardiovascular health outcomes. Time-restricted fasting (TRF) is a dietary intervention that involves limiting the time window for food consumption. It has gained attention for its potential benefits on metabolic health and weight management. This study aims to investigate the impact of TRF on key risk factors, including body weight, glucose metabolism, blood pressure, and lipid profile.

**Methods:**

We conducted a systematic search in five databases (Scopus, Embase, PubMed, Cochrane, and Web of Science) for relevant studies up to January 2023. After applying inclusion criteria, 12 studies were eligible for analysis. Quality assessment was conducted using the ROB-2.0 tool and ROBINS-I. Risk of bias was mapped using Revman 5.3, and data analysis included Hartung-Knapp adjustment using R 4.2.2.

**Results:**

The group that underwent the TRF intervention exhibited a significant decrease in body weight (SMD: -0.22; 95%CI: -0.41, -0.04; *P <* 0.05) and fat mass (SMD: -0.19; 95%CI: -0.36, -0.02; *P <* 0.05), while maintaining lean mass (SMD: -0.09; 95%CI: -0.08, 0.26; *P >* 0.05).

**Conclusion:**

TRF has shown potential as a treatment strategy for reducing total body weight by targeting adipose tissue, with potential improvements in cardiometabolic function.

**Supplementary Information:**

The online version contains supplementary material available at 10.1186/s12872-024-03863-6.

## Introduction

Cardiovascular disease (CVD) is a non-communicable disease that causes over 17.3 million premature deaths annually, making it a prominent global cause of mortality. Projections suggest that by 2030, CVD-related mortality may exceed 23.6 million [1, 2]. Studies have demonstrated an uneven distribution of cardiovascular disease mortality, with over three-quarters of deaths occurring in low- and middle-income countries [3]. Based on cardiovascular disease model projections, China currently faces a burden of over 330 million cardiovascular disease cases, with approximately 245 million cases attributed to hypertension alone.The etiology of this situation is multifactorial [4]. Prevention plays a crucial role in mitigating the risk of future cardiovascular disease development among individuals at high risk [5]. Modifying dietary behavior is a crucial strategy for preventing primary and secondary cardiovascular events [6]. Intermittent fasting is a health-promoting dietary pattern characterized by alternating between periods of eating and extended fasting. It has gained recognition for its potential benefits in weight loss and improving body composition [7]. In contrast, time-restricted fasting stands as one of the most promising approaches in intermittent energy restriction protocols. This method involves limiting the daily eating window to 4–10 h over several consecutive weeks [8]. However, there is a paucity of prospective studies investigating the potential benefits of time-restricted fasting. Meta-analyses provide objective and comprehensive evidence in the field of medical research [9]. The aim of this study is to conduct a systematic review and meta-analysis to investigate the effects of time-restricted fasting on metabolic parameters in adults, including body weight, glucose metabolism, blood pressure, and lipid profile. The study aims to provide a comprehensive analysis of the potential benefits of time-restricted fasting in improving cardiovascular health outcomes. The systematic review and meta-analysis aim to synthesize existing evidence, identify research gaps, and provide insights for future studies and clinical practices to advance the field of preventive cardiovascular medicine.

## Materials and methods

### Protocol and registration

The meta-analysis (CRD42022373942) evaluating the effects of time-restricted fasting on cardiovascular disease was undertaken according to the Preferred Reporting Items for Systematic Reviews and Meta-Analyses guidelines and was registered on PROSPERO [10].

### Search strategy

Literature was extracted by three researchers from five databases: Scopus, Embase, PubMed, Cochrane, and Web of Science. The searches were conducted on January 5, 2023, using identical terms to ensure accuracy. Data were independently extracted using predefined tables and any discrepancies were resolved through discussion.The search terms included mainly ‘Cardiovascular Disease’, ‘time-restricted feeding’, ‘time-restricted diet’, or ‘time-restricted eating’ (Supplementary Table 1).

### Study selection

**Fig. 1:**
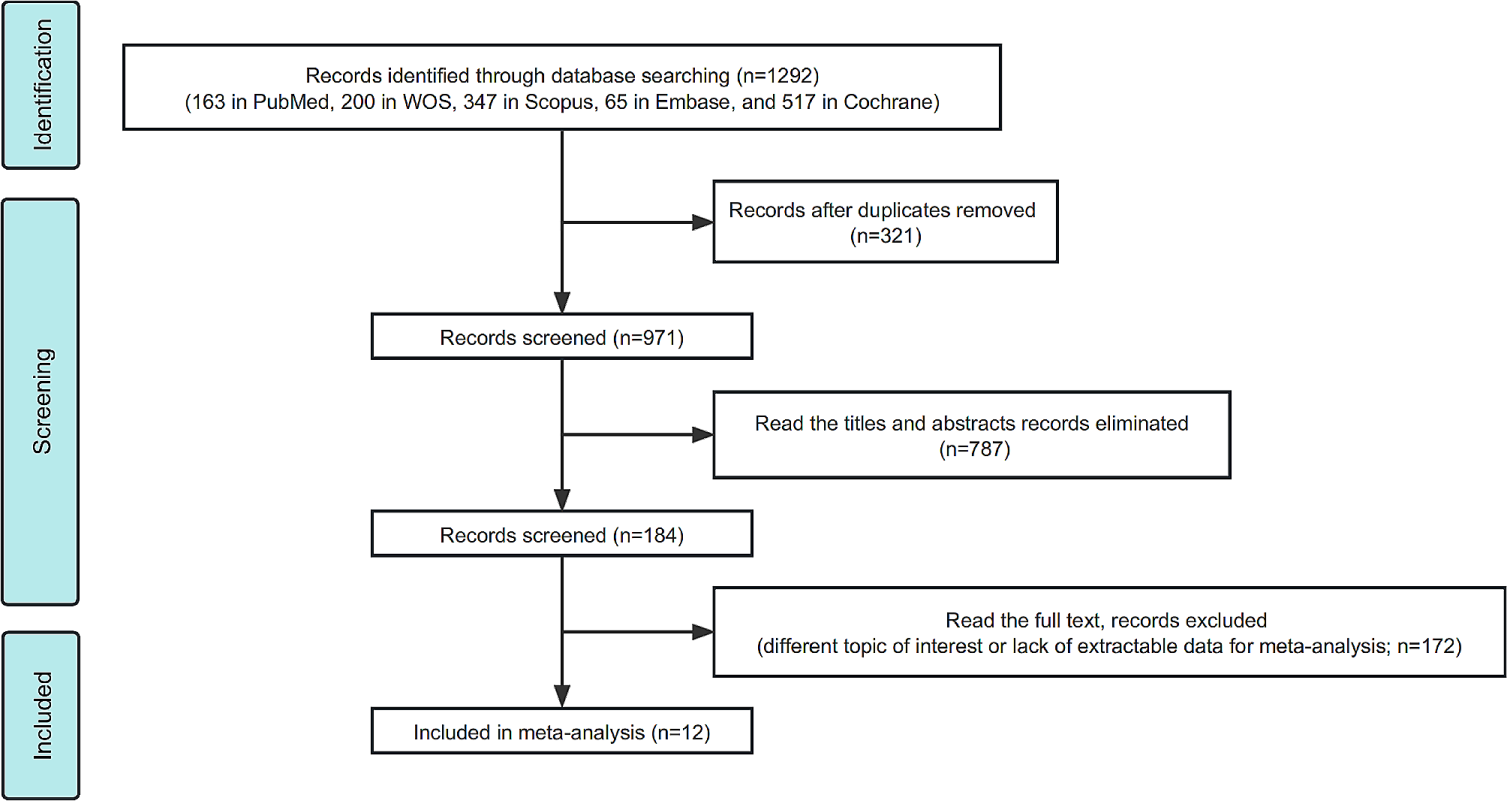
Schema of the search strategy: The figure illustrates the systematic search strategy employed to identify relevant studies for the meta-analysis

The meta-analysis included all studies, regardless of their duration. The control group participants in the included studies followed ad-libitum diets. The inclusion criteria for the studies were as follows: (1) Population: adults aged 18 years or older; (2) Intervention: fasting for 12–20 h per day; (3) Comparator: controls in randomized controlled trials (RCTs) or non-randomized controlled trials; (4) Outcome: change in at least one of the following parameters: weight, blood pressure, fasting glucose, triglycerides, low-density lipoprotein (LDL) or high density lipoprotein(HDL); (5) statistical approach to data: detailed data used to construct forest plots of continuous variables to calculate the combined results. The study included 12 articles that met the screening criteria, which excluded experiments on non-human animals, literature without valid data such as mean and standard deviation, and literature in the form of abstracts, case reports, or reviews (Fig. 1).

### Quality assessment

The revised Cochrane risk of the bias assessment tool, Risk of Bias 2.0 (ROB-2.0), was used to assess the quality of the literature on randomized controlled trials [11]. The Risk of Bias in Non-Randomized Studies of Interventions (ROBINS-I) tool was utilized to evaluate the quality of the literature for non-randomized controlled trials [12]. Additionally, Revman 5.3 was employed to map the risk of bias [13]. Furthermore, inter-rater agreement for quality assessment was determined by calculating the kappa statistics between the authors ensuring consistency and reliability in the quality assessment process [14].

### Statistical analysis

Revman 5.3 was used to conduct a meta-analysis of the extracted literature data. Cone plots were employed to determine homogeneity. Metafor package in R was used to perform Egger’s test to detect publication bias (*P* < 0.05) [15]. The literature data were recorded as standardized mean differences (SMD) for comparison of mean differences of different magnitudes. The association of time-restricted fasting with selected cardiovascular influences was assessed using a 95% CI. The model was selected based on the value of I^2^. If I^2^ was greater than 40%, a random-effects model was used to account for possible heterogeneity. If I^2^ was less than 40%, a fixed-effects model was used. The metafor package in R was used to perform Hartung-Knapp adjustments for the meta-analysis. Subsequently, the adjusted meta-analytic effect size was interpreted and analyzed.

## Results

### Literature search results

A total of 1292 articles were retrieved by searching the databases using the search strategy. After removing 321 duplicates, 971 articles remained. Finally, 12 studies were included in the study. The literature search process is depicted in Fig. 1.

### Study characteristics

The study included 479 participants, 152 of whom were men and 327 of whom were women. Table 1 summarizes the demographic and clinical characteristics of the participants.

**Table 1:** Baseline levels of subjects in the 12 studies included in the meta-analysis

Study	Participants	Study Design	Diet length	TRE Regimen (Fasting: Feeding)	Number of participants (comparator/control)	Plasma lipids (mmol/L)	Fasting Glucose (mmol/L)	Age (Year)	Sex (Female/Male)	Body Composition	Blood Pressure (mmHg)
Andriessen 2022	Adults with type 2 diabetes	RTC	3 weeks	14:10	14/14	N/A	7.9 ± 1.3	67.5 ± 5.2	7 F/7 M	BMI:30.5 ± 3.7	N/A
Anton 2019	Overweight, sedentary older adults	One group pretest–posttest design	4 weeks	16:08	10/10	N/A	5.87 ± 1.57	≥ 65	6 F/4 M	BMI:34.1 ± 3.3; WC:109.43 ± 12.9 cm	SBP:145.9 ± 15.6 DBP:78.1 ± 12.4
Brady 2021	Middle and long-distance runners	RTC	8 weeks	16:08	10/7	N/A	N/A	36.4 ± 7.4	17 M	Body mass:75.6 ± 9.3 kg; Fat mass:12.4 ± 4.3 kg; Height:1.79 ± 0.06 m	N/A
Cai 2019	NAFLD patients	RTC	4 weeks/12 weeks	16:08	95/79	TG:2.65 ± 1.69; LDL:2.55 ± 0.79; HDL:1.16 ± 0.50	5.09 ± 0.90	34.54 ± 6.96	145 F/52 M	BMI:26.34 ± 2.73; Fat mass:29.06 ± 3.64 kg Lean mass:43.65 ± 3.95 kg; WC:92.59 ± 4.98 cm	N/A
Chiu 2022	Healthy male adults	RTC	5 days	16:08	8/8	TG:1.45 ± 0.70	3.94 ± 0.18	22 ± 1.3	8 M	BMI:26.0 ± 0.38	N/A
Chow 2020	Overweight adults	RTC	12 weeks	16:08	11/12	TG:0.98 ± 0.24	5.06 ± 0.72	45.5 ± 12.1	17 F/3 M	BMI 34.1 ± 7.5; Fat mass:45.6 ± 20.7 kg; Lean Mass:51.1 ± 8.7 kg	SBP:123 ± 13 DBP:79 ± 8
Correia 2021	Healthy male physical education students	RTC	30 days	16:08	12/12	N/A	N/A	22.4 ± 2.8	12 M	BMI:24.2 ± 2.0; Body mass: 73.6 ± 9.5 kg	N/A
Kesztyüs 2021	Healthy adults	One group pretest–posttest design	3 months	16:08	61/63	TG:1.31 ± 0.66; HDL:1.74 ± 0.39; LDL:3.48 ± 0.89	N/A	47.8 ± 10.5	53 F/8 M	BMI:26.1 ± 4.6 ;WC:89.1 ± 12.0 cm	N/A
Lin 2022	Middle-aged women	RTC	8 weeks	16:08	30/33	TG:1.22 ± 0.53; HDL:1.64 ± 0.31; LDL:2.79 ± 0.68	4.97 ± 0.48	54.2 ± 7.9	65 F	BMI:25.7 ± 3.8 ;WC:89.7 ± 9.5 cm	SBP:121.1 ± 12.9 DBP:71.2 ± 10
Moro 2016	Resistance trained males	RTC	8 weeks	16:08	17/17	TG:1.52 ± 0.18; HDL:1.40 ± 0.26; LDL:3.00 ± 0.30	5.34 ± 3.63	29.21 ± 3.8	34 M	Body mass:84.6 ± 6.2 kg; Fat mass:11.05 ± 4.27 kg	N/A
Peeke 2021	Adults with obesity	Two group pretest–posttest design	8 weeks	14:10; 12:12	30/30	N/A	5.64 ± 1.09	44 ± 11	26 F/4 M	BMI:38.9 ± 7.7	N/A
Zhao 2022	Adults with obesity	One group pretest–posttest design	8 weeks	14:10	15/15	TG:1.7 ± 0.6; HDL:1.06 ± 0.21	5.7 ± 0.4	63 ± 4	15 M	BMI:30.5 ± 2.4; WC:113 ± 4 cm	SBP:136 ± 19 DBP:83 ± 10

### Evaluation of the quality of the studies

**Fig. 2:**
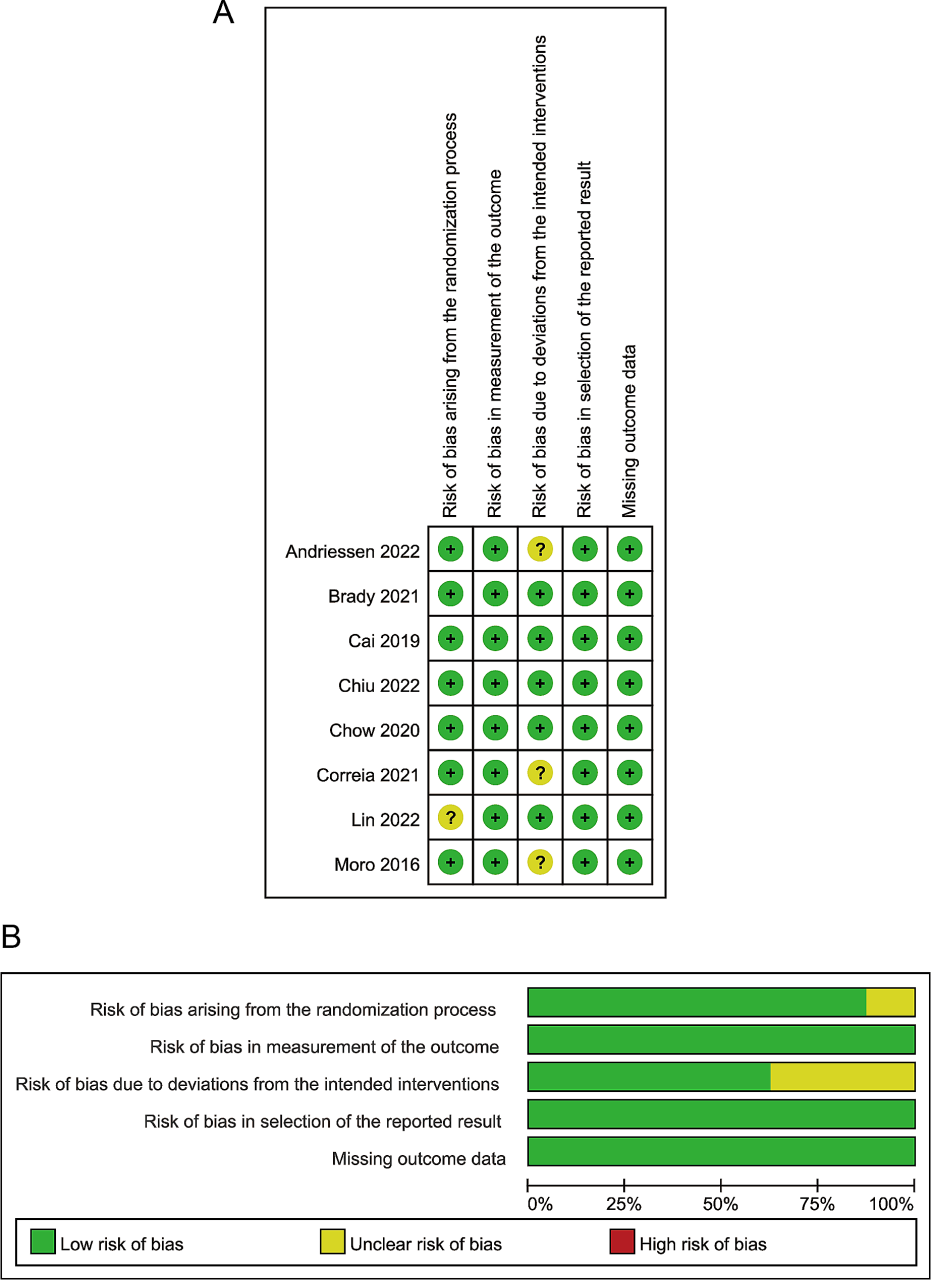
Risk of bias graph for literature quality (RCT): The graph presents the assessment of the risk of bias in randomized controlled trials (RCTs) included in the meta-analysis

**Fig. 3:**
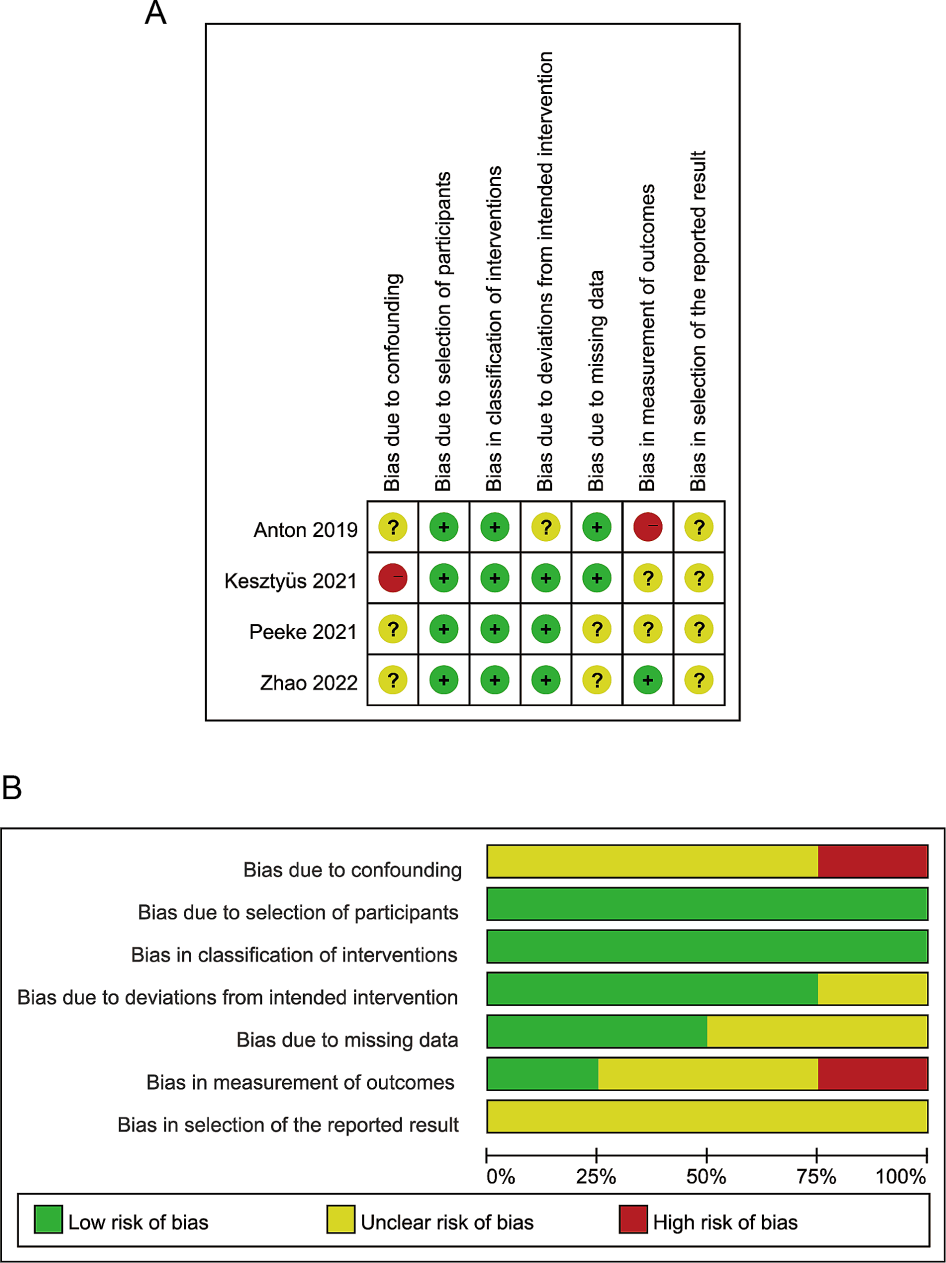
Risk of bias graph for literature quality (non-RCT): The graph displays the assessment of the risk of bias in non-randomized controlled trials (non-RCTs) included in the meta-analysis

The quality of the 12 selected studies was evaluated using the ROB-2.0 and ROBINS-I evaluation tools [16–27]. The quality assessment results are presented in Figs. 2 and 3. Overall, the included studies exhibited a low risk of bias and were considered suitable. The 12 studies were evaluated by three assessors using Fleiss’ Kappa value, resulting in a score of 0.775, indicating a high level of agreement among the assessors. Additionally, the z-value of 4.65 and the *p*-value of 3.32e-06 (much less than 0.05) suggest that inter-assessor agreement is significantly higher than random agreement.

### Effects of time-restricted fasting on body weight and body composition

**Fig. 4:**
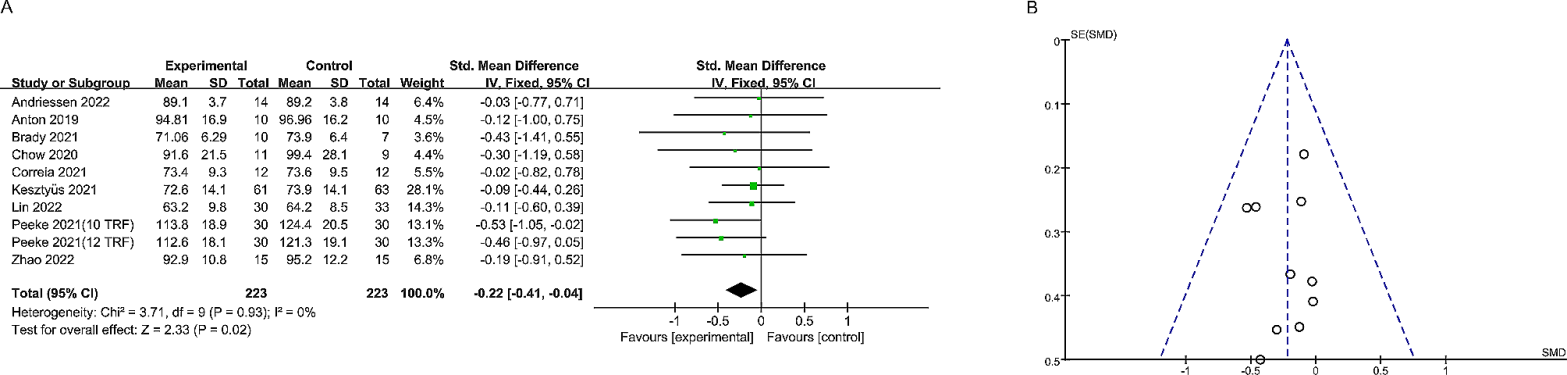
The forest plot (**A**) and funnel plot (**B**) summarize the effect of time-restricted fasting on body weight compared to the control group: The forest plot (**A**) visually demonstrates the effect size of time-restricted fasting on body weight, while the funnel plot (**B**) assesses publication bias

**Fig. 5:**
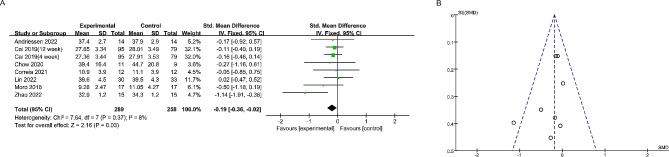
The forest plot (**A**) and funnel plot (**B**) summarize the effect of time-restricted fasting on fat mass compared to the control group: The forest plot (**A**) visually represents the effect size of time-restricted fasting on fat mass, while the funnel plot (**B**) evaluates publication bias

**Fig. 6:**
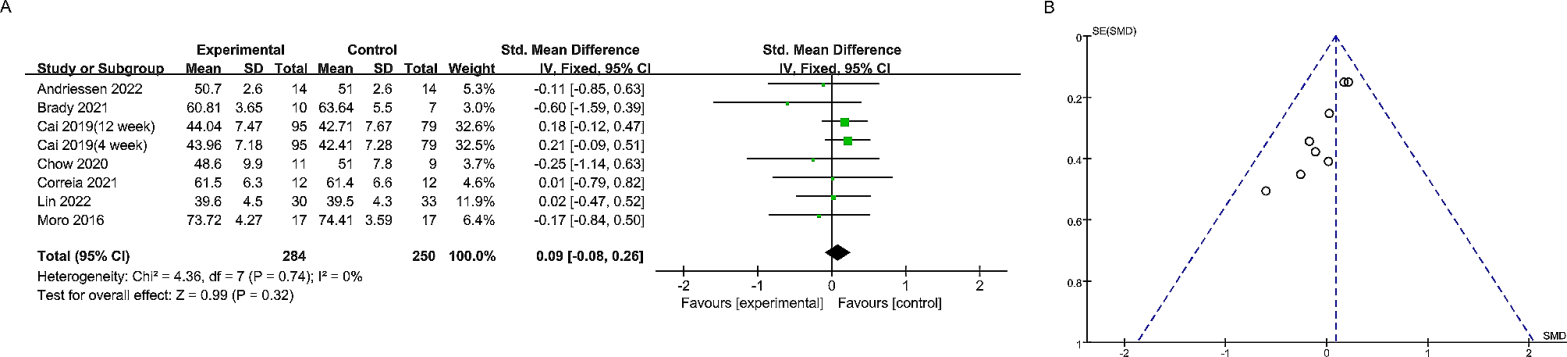
The forest plot (**A**) and funnel plot (**B**) summarize the effect of time-restricted fasting on lean mass compared to the control group: The forest plot (**A**) depicts the effect size of time-restricted fasting on lean mass, while the funnel plot (**B**) examines publication bias

Nine studies reported the effect of time-restricted fasting on body weight (Fig. 4). A fixed effects model revealed an SMD of -0.22 (95% CI: -0.41, -0.04), indicating a significant weight loss effect, and an I^2^ of 0, indicating no significant heterogeneity between studies. Additionally, the symmetrical funnel plot and non-significant Egger’s test (*P* > 0.05) suggested the absence of publication bias. Seven studies reported alterations in body composition. The study found that time-restricted fasting significantly reduced the amount of fat in the subjects, with no significant heterogeneity between studies (SMD: -0.19; 95% CI: -0.36, -0.02) (Fig. 5). Additionally, no significant publication bias was observed (Egger’s test: *P* > 0.05). Notably, Fig. 6 shows that time-restricted fasting did not affect the lean body mass of the subjects, despite the varying duration of the intervention (SMD: -0.09; 95% CI: -0.08, 0.26). The results of the Egger test are shown in Supplementary Fig. 1. After applying the Hartung-Knapp adjustment and comparing the data, the weight loss trend in the test group was no longer statistically significant (*P* = 0.10). However, the conclusion that there was a significant reduction in body fat (*P* < 0.01) while muscle mass remained unchanged (*P* > 0.05) remains valid(Table 2; Supplementary Table 2).

**Table 2:** Meta-analysis of Cardiometabolic Parameters

Outcome	Number of studies	SMD (95%CI)	Heterogeneity I^2^%
Fasting glucose	10 [27–36]	-0.18 [-0.44, 0.08]	58
Triglycerides	9 [28–30, 32–37]	-0.03 [-0.26, 0.21]	48
HDL	6 [28–30, 32, 34, 37]	-0.05 [-0.21, 0.11]	19
LDL	5 [28, 29, 32, 34, 37]	-0.05 [-0.35, 0.26]	72
Systolic blood pressure	4 [27–30]	-0.22 [-0.56, 0.13]	0
Diastolic blood pressure	4 [27–30]	-0.28 [-0.63, 0.07]	0

### Effects of time-restricted fasting on cardiometabolic parameters

Nine studies investigated the effect of time-restricted fasting on triglycerides, six reported its effect on HDL, five on LDL, and ten on fasting glucose. Additionally, four studies investigated the effect of time-restricted fasting on systolic and diastolic blood pressure. Although there was a trend towards a decrease in almost all cardiometabolic parameters, the time-restricted fasting intervention did not significantly reduce these parameters compared to the control group(Table 2; Supplementary Fig. 2). Following the Hartung-Knapp adjustment, the experimental group exhibited a significant decrease in systolic blood pressure(*P* = 0.01). However, there were no significant differences observed in the remaining data, including fasting glucose, triglycerides, HDL, LDL, and diastolic blood pressure (Supplementary Table 2).

## Discussion

The study suggests that time-restricted fasting interventions can effectively reduce body weight and fat mass while retaining lean body mass. This indicates that subjects lost fat rather than muscle and water. When the calculation method was modified, for example, by introducing the Hartung-Knapp adjustment, the results showed slight differences. Although the decrease in body fat percentage remained statistically significant, there was no significant difference in weight loss. However, it is important to note that a significant decrease in systolic blood pressure was observed in this context.

The weight loss observed during time-restricted fasting primarily results from the energy imbalance it creates. By narrowing the eating window, overall caloric intake is frequently reduced, leading to a negative energy balance [28]. This compels the body to utilize stored fat as an energy source, resulting in weight loss [29]. Additionally, time-restricted fasting has the potential to enhance metabolic efficiency and fat oxidation, which further contributes to weight reduction [30]. Simultaneously, time-restricted fasting may potentially contribute to a decrease in systolic blood pressure. The exact mechanism behind this effect is not entirely clear, but it could be associated with improvements in metabolic parameters, including insulin resistance and inflammation, both of which are significant contributors to the development of hypertension [31]. Furthermore, it is worth noting that weight loss is correlated with a decrease in blood pressure, indicating that the decline in systolic blood pressure may be a secondary effect of weight loss [32, 33].

The studies included in the analysis indicate that time-restricted fasting did not result in significant improvements in certain cardiovascular health indicators, such as fasting glucose, triglycerides, HDL, LDL, and diastolic blood pressure. The lack of improvement may be attributed to various factors, including the presence of confounding variables that may have influenced the outcomes. Confounding factors, such as variations in dietary habits among different regions where time-restricted fasting was implemented, might have influenced the results [34]. It is possible that time-restricted fasting alone did not directly affect the dietary content of the participants, which may have led to limited changes in cardiovascular health indicators. Future studies should consider controlling for dietary factors to isolate the effects of time-restricted fasting on cardiovascular health indicators. Longer intervention periods may be necessary to observe substantial improvements in certain indicators. Additionally, the lack of significant improvements may be attributed to the gender composition of the enrolled population. Cardiovascular health can differ between males and females, and considering this aspect can provide valuable insights into potential gender-specific effects of time-restricted fasting. Future studies should aim to recruit a balanced representation of both genders to assess whether the impact of time-restricted fasting varies based on sex [35]. Clinical trials on time-restricted fasting typically involve adults with physical and biochemical abnormalities, such as diabetes, metabolic syndrome, and obesity. Most of the trials conducted on time-restricted fasting have been short-term interventions. As a result, there is limited long-term data on the effects of this practice on children, pregnant women, the elderly, and people with chronic diseases. Additionally, daily restricted diets may not be practical for individuals who have frequent evening business meetings or those who are accustomed to enjoying dinner with their families. As an alternative, the ‘5 + 2’ fasting model may be more feasible. It is important to note that although no adverse effects have been reported, the long-term effects of transitioning from three meals to two meals per day on the digestive system are still unknown [36].

Both physiological and psychological issues require sufficient attention [37]. If the daily feeding period is too short, participants may experience negative effects on their mental health by suppressing their appetite for an extended period to maintain sufficient fasting length [38]. Currently, there is limited data on the efficacy of time-restricted fasting in modulating human health. Only a dozen studies have examined the effects of such diets on human body weight and other metabolic disease risk parameters. Although these findings provide important preliminary evidence, they are limited in several ways. All the analyzed studies had short observation periods. While various factors, including adherence issues, may affect longer-term outcomes, it is important to consider the evidence from longer-term trials [39]. Furthermore, the small sample size in each study may significantly limit the detection of secondary outcome indicators [40, 41]. Some of these trials employ a crossover design, which may not be appropriate when expecting a change in health status. This is because subjects may not return to their baseline weight before a new intervention period begins, which could affect the trial results. No trials have yet examined whether time-restricted fasting can maintain weight loss. Future trials with long parallel arms (over 6 months) should be conducted to test this hypothesis. Additionally, sample sizes should be expanded, and direct comparisons of time-restricted fasting with other weight loss methods should be made to determine the efficacy of maintaining weight loss once reliable conclusions are drawn.

The evidence suggests that a time-restricted fasting program intervention leads to a slight reduction in weight (1-4% from baseline levels) in overweight and obese individuals [42]. Shorter meal times (4–6 h) do not result in more discomfort compared to longer meal times (8–10 h). Weight loss achieved through time-restricted fasting primarily originates from a reduction in fat mass rather than lean mass or water, which is crucial for preventing cardiovascular disease. Time-restricted fasting offers the advantage of a fixed daily fasting period that creates an energy deficit of 350–500 calories. This eliminates the necessity to calculate daily calorie intake and provides improved control. Accurately tracking long-term calorie intake can be challenging [43]. Additionally, time-restricted fasting may reduce blood pressure and oxidative stress markers to some extent [44]. The impact of this fasting regimen on plasma lipids is unclear. Some studies indicate a decrease in LDL cholesterol and triglycerides, while others show no effect. Based on current findings, time-restricted fasting does not seem to affect HDL cholesterol levels, but it does appear to decrease inflammatory markers such as CRP, homocysteine, IL-6, or TNF-α. Few adverse events were reported during time-restricted fasting, indicating that the diet is generally safe for human subjects.

## Conclusion

In summary, our research shows that time-restricted fasting effectively reduces body weight and fat mass without affecting lean body mass, indicating a preference for fat loss. This finding highlights the potential of time-restricted fasting as a strategy for improving body composition. However, further clinical trials are needed to explore the underlying mechanisms and broader impacts of this intervention, particularly in relation to cardiovascular health. Future research should aim to clarify gender-specific responses to time-restricted fasting, considering differences in adipose tissue characteristics and metabolism. This will enhance our understanding of the role of time-restricted fasting in promoting metabolic health.

### Electronic supplementary material

Below is the link to the electronic supplementary material.

Supplementary Material 1

## Data Availability

The data used and/or analyzed during the current study are available from the corresponding author upon request.

## Abbreviations

CVD: Cardiovascular Disease
TRF: Time-restricted Fasting
PROSPERO: International Prospective Register of Systematic Reviews
RCT: Randomized Controlled Trial
ROB-2.0: Risk of Bias 2.0
ROBINS-I: Risk Of Bias In Non-randomized Studies of Interventions
Revman 5.3: Review Manager version 5.3
SMD: Standardized Mean Difference
CI: Confidence Interval
CRP: C-Reactive Protein
IL-6: Interleukin-6
TNF-α: Tumor Necrosis Factor Alpha
HDL: High-Density Lipoprotein
LDL: Low-Density Lipoprotein
