# Supplementary material for: Beneficial effects of time-restricted fasting on cardiovascular disease risk factors: a meta-analysis
Source: BMC Cardiovasc Disord. 2024 Apr 16;24:210. doi: 10.1186/s12872-024-03863-6 (PMC11020908; doi:10.1186/s12872-024-03863-6)
Supplement: Supplementary file 1 — Supplementary Material 1 [file 12872_2024_3863_MOESM1_ESM.docx]

Supplementary Materials

**Table S1: Search Strategy**

| Literature Sources | Search Strategy |
| --- | --- |
| Cochrane | ID Search Hits  #1 MeSH descriptor: [Fasting] explode all trees 3507  #2 (Intermittent Fasting or Fasting, Intermittent or Intermittent Fastings or Hunger Strike or Hunger Strikes or Strike, Hunger or Strikes, Hunger or Time Restricted Feeding or Feeding, Time Restricted or Time Restricted Feedings):ti,ab,kw (Word variations have been searched) 1402  #3 #1 or #2 4791  #4 MeSH descriptor: [Cardiovascular Diseases] explode all trees 118663  #5 (Cardiovascular Disease or Disease, Cardiovascular or Diseases, Cardiovascular):ti,ab,kw (Word variations have been searched) 52391  #6 #4 or #5 153159  #7 #3 and #6 517 |
| Embase | No. Query Results  #3. #1 AND #2  #2. 'intermittent fasting':ab,ti OR 'fasting,  intermittent':ab,ti OR 'intermittent  fastings':ab,ti OR 'hunger strike':ab,ti OR  'hunger strikes':ab,ti OR 'strike, hunger':ab,ti  OR 'strikes, hunger':ab,ti OR 'time restricted  feeding':ab,ti OR 'feeding, time  restricted':ab,ti OR 'time restricted  feedings':ab,ti  #1. 'cardiovascular disease':ab,ti OR 'disease  cardiovascular':ab,ti OR 'diseases  cardiovascular':ab,ti |
| Pubmed | (((("Cardiovascular Diseases"[Mesh]) OR (Cardiovascular Disease[Title/Abstract])) OR (Disease, Cardiovascular[Title/Abstract])) OR (Diseases, Cardiovascular[Title/Abstract])) AND ((((((((((Intermittent Fasting[Title/Abstract]) OR (Fasting, Intermittent[Title/Abstract])) OR (Intermittent Fastings[Title/Abstract])) OR (Hunger Strike[Title/Abstract])) OR (Hunger Strikes[Title/Abstract])) OR (Strike, Hunger[Title/Abstract])) OR (Strikes, Hunger[Title/Abstract])) OR (Time Restricted Feeding[Title/Abstract])) OR (Feeding, Time Restricted[Title/Abstract])) OR (Time Restricted Feedings[Title/Abstract])) |
| WOC | (TS=("Cardiovascular Diseases") OR AB=((Cardiovascular Disease) OR (Disease, Cardiovascular) OR (Diseases, Cardiovascular))) AND (TS=((Intermittent Fasting) OR (Fasting, Intermittent) OR (Intermittent Fastings) OR (Hunger Strike) OR (Hunger Strikes) OR (Strike, Hunger) OR (Strikes, Hunger) OR (Time Restricted Feeding) OR (Feeding, Time Restricted) OR (Time Restricted Feedings))) |
| Scope | ( TITLE-ABS-KEY ( cardiovascular  AND disease )  OR  TITLE-ABS-KEY ( disease  AND cardiovascular )  OR  TITLE-ABS-KEY ( diseases  AND cardiovascular ) )  AND  ( TITLE-ABS-KEY ( intermittent  AND fasting )  OR  TITLE-ABS-KEY ( Fasting AND Intermittent)  OR  TITLE-ABS-KEY ( Intermittent AND Fastings)  OR  TITLE-ABS-KEY (Hunger AND Strike) OR TITLE-ABS-KEY (Hunger AND Strikes) OR TITLE-ABS-KEY (Time AND Restricted AND Feedings) OR TITLE-ABS-KEY ( Time AND Restricted AND Feeding)) |

**Table S2:Effects of time-restricted fasting on cardiovascular health markers adjusted by Hartung-Knapp method**

| Items | Model Results | | |
| --- | --- | --- | --- |
|  | p-value | Heterogeneity I2 % | tau2 |
| Body weight | 0.101 | 0.00 [0.00, 71.39] | 0.00 [0.00, 25.13] |
| Fat mass | 0.007 | 6.44 [0.00, 61.22] | 0.04 [0.00, 0.95] |
| Lean mass | 0.585 | 0.00 [0.00, 73.70] | 0.00 [0.00, 5.47] |
| Fasting glucose | 0.197 | 71.79 [36.55, 88.59] | 0.86 [0.01, 0.25] |
| Triglycerides | 0.896 | 45.84 [0.00, 75.18] | 0.01 [0.00, 0.05] |
| HDL | 0.985 | 2.49 [0.00, 94.17] | 0.00 [0.00, 0.11] |
| LDL | 0.833 | 72.96 [31.72, 94.20] | 0.05 [0.01, 0.34] |
| Systolic blood pressure | 0.011 | 0.00 [0.00, 0.00] | 0.00 [0.00, 0.00] |
| Diastolic blood pressure | 0.100 | 0.00 [0.00, 86.63] | 0.00 [0.00, 96.41] |


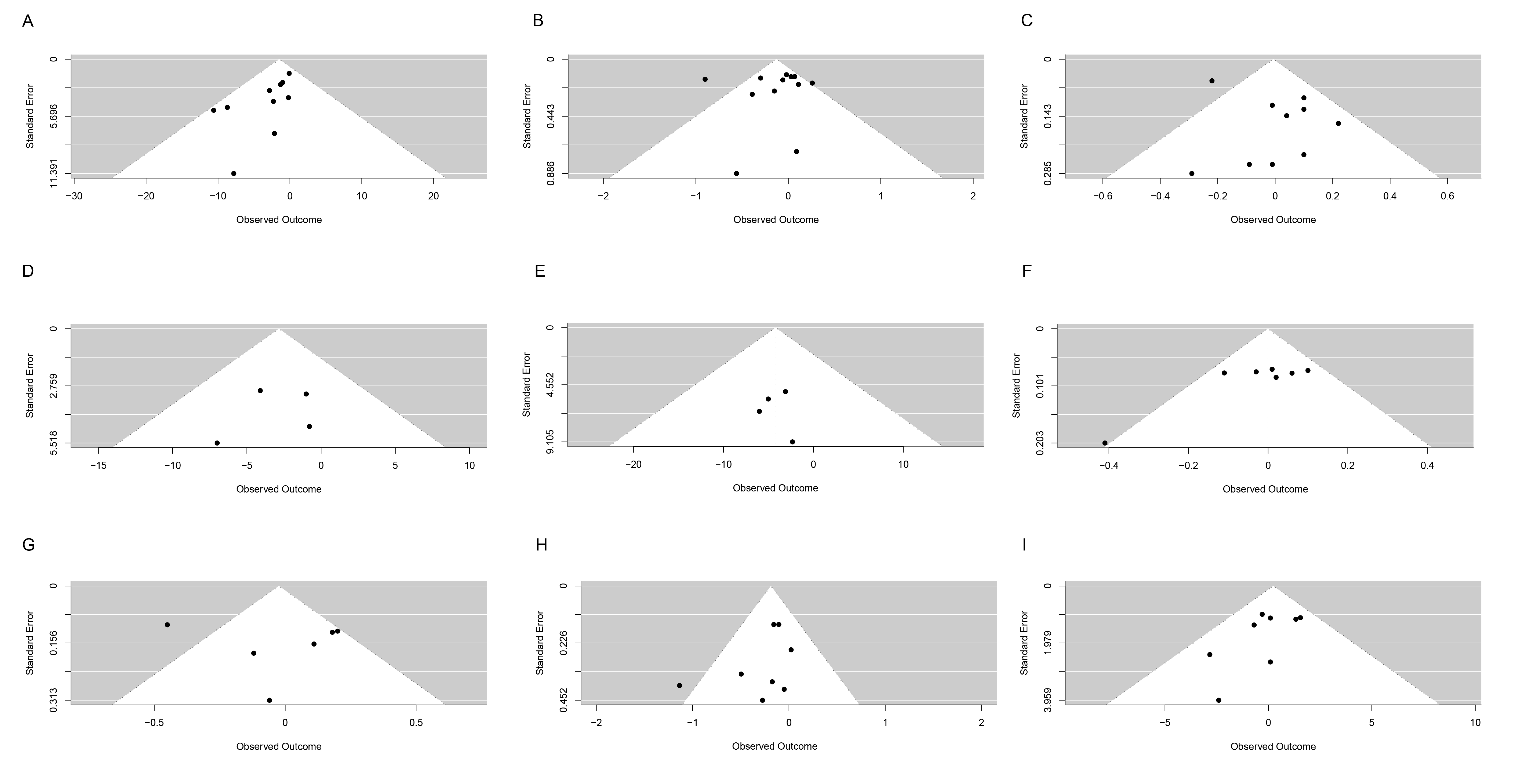


Figure S1 This figure consists of nine panels (A-I), each depicting the results of Egger's test for funnel plot asymmetry across various health-related metrics, indicating potential publication bias in meta-analyses. Panel A: Body Weight, z = -1.62 , p = 0.10; Panel B: Fasting Glucose, z = -1.62 , p = 0.10; Panel C: Triglyceride, z = -0.10 , p = 0.91; Panel D: Diastolic Blood Pressure, z = -1.50 , p = 0.12; Panel E: Systolic Blood Pressure, z = -1.35 , p = 0.17; Panel F: High Density Lipoprotein, z = -0.02, p = 0.98; Panel F: Low-Density Lipoprotein, z = -0.20 , p = 0.83; Panel H: Fat Mass, z = -1.29 , p = 0.24; Panel I: Lean Mass, z = 0.49 , p = 0.62. Overall, these analyses suggest that there is no significant evidence of publication bias in the studies included in the meta-analyses for these health-related metrics.


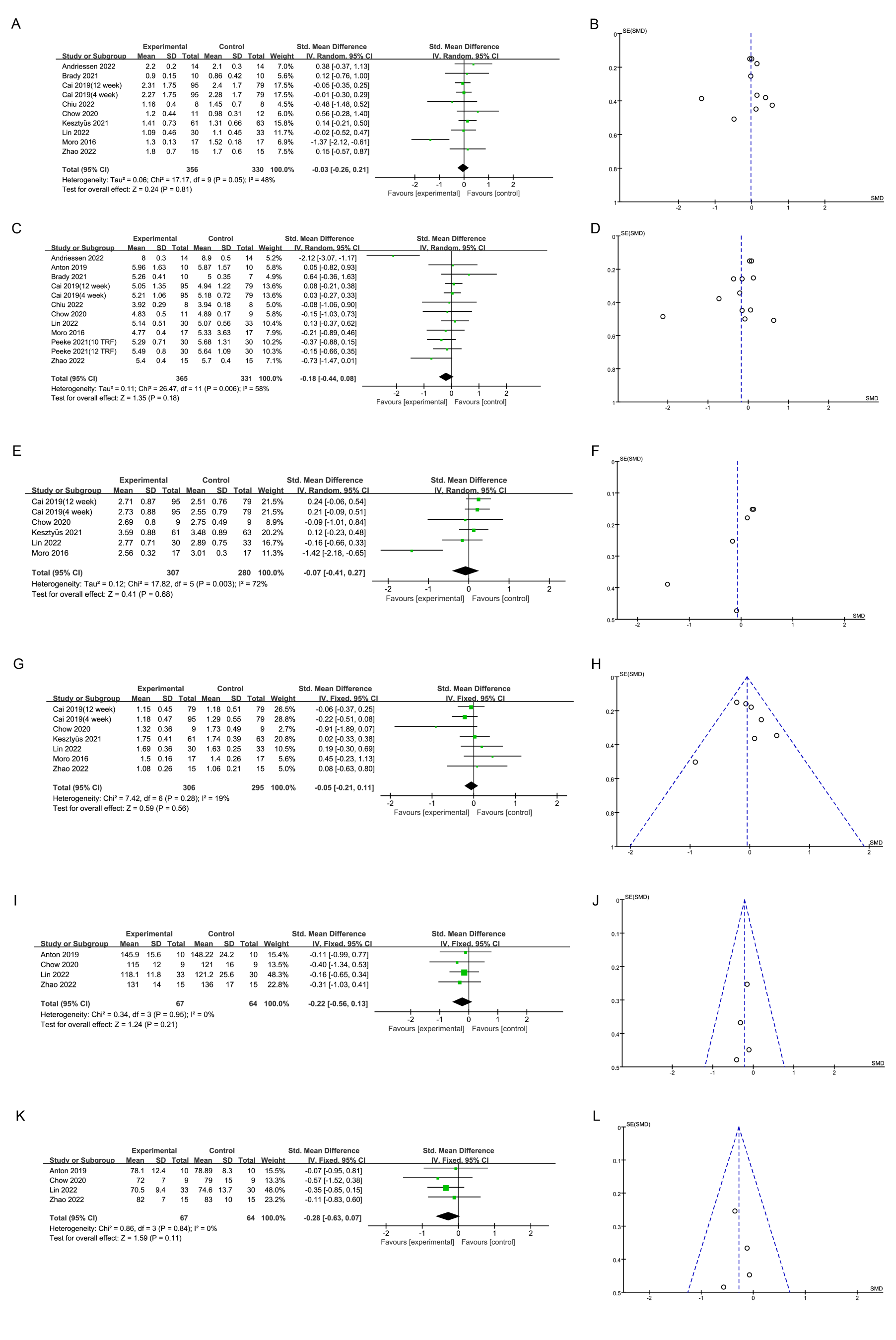


Figure S2 The forest plot and funnel plot summarize the effect of time-restricted fasting on Triglycerides (A-B), Fasting glucose (C-D), LDL (E-F), HDL (G-H), Systolic blood pressure (I-J), and Diastolic blood pressure (K-L). The forest plot (A-J) presents the effect size of time-restricted fasting on various cardiovascular disease risk factors. The funnel plot (B-L) assesses publication bias.
